# Study of an Extensive Set of Eye Movement Features: Extraction Methods and Statistical Analysis

**DOI:** 10.16910/jemr.11.1.3

**Published:** 2018-03-20

**Authors:** Ioannis Rigas, Lee Friedman, Oleg Komogortsev

**Affiliations:** Texas State University, San Marcos,, USA

**Keywords:** eye movements, feature extraction, saccades, fixations, post-saccadic oscillations, variability, test-retest reliability, factor analysis

## Abstract

This work presents a study of an extensive set of 101 categories of eye movement features from three types of eye movement events: fixations, saccades, and post-saccadic oscillations. We present a unified framework of methods for the extraction of features that describe the temporal, positional and dynamic characteristics of eye movements. We perform statistical analysis of feature values by employing eye movement data from a normative population of 298 subjects, recorded during a text reading task. We present overall measures for the central tendency and variability of feature values, and we quantify the test-retest reliability of features using either the Intraclass Correlation Coefficient (for normally distributed and normalized features) or Kendall’s coefficient of concordance (for non-normally distributed features). Finally, for the case of normally distributed and normalized features we additionally perform factor analysis and provide interpretations of the resulting factors. The presented methods and analysis can provide a valuable tool for researchers in various fields that explore eye movements, such as in behavioral studies, attention and cognition research, medical research, biometric recognition, and humancomputer interaction.

## Introduction

The extraction of eye movement features for modeling the structure and functionality of the oculomotor system is a vital task in many fields of research. Human eye movements can serve as an investigation tool in cognitive and behavioral studies, given their inherent connection to the guiding mechanisms of visual attention. The connections of eye movements and the performed cognitive task was systematically investigated in (1). The advances in eye-tracking technology, allowed for the adoption of eye movement analysis in studies of cognitive psychology in various fields, such as linguistics, spatial processing, reading, and problem solving (1, 2 3, ). Several research studies specifically explored the underlying mechanisms connecting eye movements with visual attention and perception (4, 5, 6). Also, the increasing affordability of mobile eye-trackers facilitated the inspection of natural human behavior in out-of-the-lab environments (7 8, ).

There are numerous studies that focused on the interconnections of oculomotor behavior and individual characteristics. Eye movements have been explored in relation to individual motivation (9), and the ‘Big 5’ personality traits (agreeableness, conscientiousness, extraversion, neuroticism, openness) (10). Recently, vigor of eye movements was associated with the personal impulsiveness during decision-making tasks (11). Additionally, the research of personal traits in oculomotor structure and functionality has served as the basis for the field of eye movement biometrics (12).

Another field of use of eye movements is clinical research. Irregular eye movements have been examined as indicators of pathophysiological neural abnormalities, and for the identification of early signs of neurodegenerative diseases (13). The characteristics of eye movements during reading have been investigated in research studies of early Alzheimer’s disease (14) and Parkinson’s disease (15). Furthermore, there are studies exploring the oculomotor behavior in various behavioral disorders, such as ADHD (16) and autism (17 18, ).

The research on the extraction of eye movement features has been fragmented, since most eye movement studies focus on small sets of features related each time to aspecial topic under consideration. This motivated our current study on the extraction and analysis of an *extensive* set of eye movement features, from fixations, saccades, and post-saccadic oscillations. For our analysis, we use data recorded during the task of reading. Such a task allows for the extraction of a large diversity of features that can be used to describe physiological and behavioral properties of eye movements. 

The contribution of our current work can be summarized as follows:

We present methods for the extraction of an extensive collection of 101 general categories of eye movement features from pre-classified eye movement events (fixations, saccades, and post-saccadic oscillations). Code and data for the extraction of features are publicly available at the following link: https://digital.library.txstate.edu/handle/10877/6904
We employ data from a large database of 298 subjects recorded during a text reading task,in order to demonstrate normative values of central tendency (median) and overall variability (inter-quartile range) of the extracted features.
We evaluate the test-retest reliability of the extracted features by using measures of absolute agreement, specifically, we use the Intraclass Correlation Coefficient (ICC) for normally distributed and normalized features, and the Kendall’s coefficient of concordance (W) for non-normally distributed features.
We perform factor analysis with varimax rotation on normally distributedand normalized features, and we provide an interpretation of the resulting factors based on the most heavily weighted features contributing to each factor.

## Extraction of Eye Movement Features

### General Overview and Used Notation during Feature Extraction

Prior to feature extraction, the raw eye movement recordings (horizontal and vertical positional signal in degrees of visual angle) are preprocessed in order to classify the signal into parts corresponding to basic types of eye movement events, namely, fixations, saccades, and post-saccadic oscillations (see definitions in respective sections). The algorithm used to perform eye movement classification is a modified version of the velocity-based method presented in (19). The modifications focus on the adoption of thresholds and parameters that lead to optimum classification performance for the data of our reading text experiment. The accuracy of the algorithm was complementarily verified via visual screening of classified eye movement events.

The extracted features generally fall in one of two categories: *single-value features* and *multi-value features*. For single-value features, a unique value is calculated for each recording by applying a collective model over the values from the instances of an event-type (fixation, saccade or post-saccadic oscillation). For multi-value features, six descriptive statistics are used to model the distributions offeature values extracted from all instances of an event-type in a recording, thus generating six respective feature subtypes. The used descriptive statistics are: the mean (*Mn*), median (*Md*), standard deviation (*Sd*), interquartile range (*Iq*), skewness (*Sk*), and kurtosis (*Ku*). The features are extracted from horizontal, vertical and/or radial profiles (the term *profile* refers to the variation of a quantity –position, velocity, acceleration– in time/sample domain), or from 2-D trajectory in space.

In *List 1*, we present various symbols and notation that will be used in the descriptions of feature extraction methods in following sections.

**List 1.**
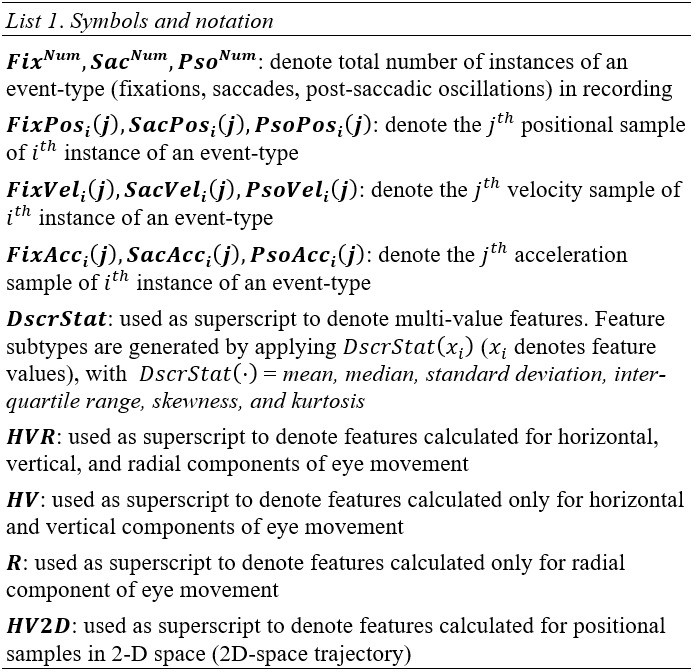


### Fixation Features

The term fixation is used to define the state when the eyes are focused on a specific area of interest, projecting the content of this area on the high-resolution processing region of the retina (fovea centralis). During fixation the eyes are not totally still but they perform various miniature movements: slow ocular drifts, small saccades (micro-saccades), and high-frequency tremors (sometimes referred as physiological nystagmus) (20). In next subsections, we describe different categories of features that can be extracted to represent temporal, positional, and dynamic characteristics of the eye movement signal during fixations.

#### Features of fixation temporal characteristics

**Figure 1.**
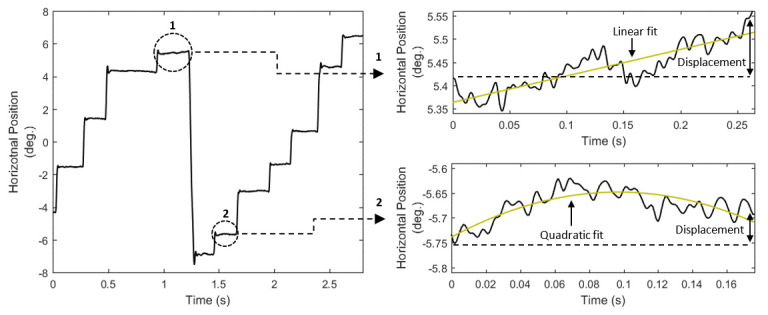
Eye movement positional signal, and examples of different ways of modeling fixation drifts (right top: linear fit is preferred; right bottom: quadratic fit is preferred).

The duration and rate of fixations (*F01*-*F02*, *List 2*) are two basic fixation features that describe the temporal behavior of the oculomotor system. For example, during reading, these characteristics can be used to examine cognitive functions co-modulated by various aspects, such as the context (21), subject-related idiosyncrasies (22), and mental workload (23). In *Figure 1* (*left*), we can overview a sequence of fixations performed during reading, and we can observe the variability in their durations.

#### Features of fixation position and drift

A simple way to model the overall fixated position is to calculate the centroid (*F03*, *List 3*) of samples in the fixation position profile. However, this simple feature cannot model the characteristics of fixation drift, i.e. the slow movement of the eye around a fixated location. Computational modeling of fixation drift can provide information about the stability of visual input in retina and the related cognitive implications (24), and also, properties of fixation drift could be used as cues for the detection of pathological conditions like amblyopia (25) and cerebellar disease (26). It should be mentioned that fixation drift can be also attributed to device dependent sources (sometimes called ‘baseline drift’), and so, the modeling of fixation drift can be particularly useful for human-computer interaction applications (27) and during the inspection of eye-tracking quality (28). Fixation drift can be manifested in various forms (*Figure 1*, *right*), and for this reason we present a number of alternative features that can be used to model the characteristics of fixation drift (*F04* to *F13*, *List 3*).

#### Features of fixation velocity and acceleration

**Figure 2.**
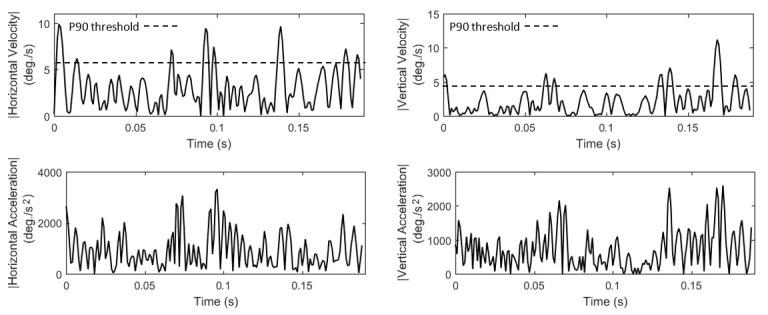
Examples of fixation velocity and acceleration profiles for horizontal and vertical components of eye movement (P90 denotes the 90-th percentile velocity threshold).

Due to limited eye mobility during fixations, their velocity and acceleration profiles are usually affected by noise. However, the variability in these profiles can also reflect information stemming from physiological sources, e.g., micro-movements and oculomotor function irregularities. Such movements have been explored in studies of visual perception (29), or for the examination of pathological conditions (30,31, ). In *Lists 4-5* we present the features that model fixation velocity and acceleration (*F14* to *F25*), and in *Figure 2* we show examples of fixation velocity and acceleration profiles. Most features are extracted via the statistical modeling of profiles using the mean, median, standard deviation, skewness and kurtosis. Such profile-modeling features have been previously employed in eye movement biometrics (32) both for fixations and saccades. It is important to clarify that the statistical modeling of profiles should not be confused with the mechanism used for creating feature subtypes (previously described in general overview section). The employed statistics are similar but, in the current case, they are used as the means for modeling the ‘shapes’ of profiles.

#### Lists of Fixation Features

**List 2. - List 5.**
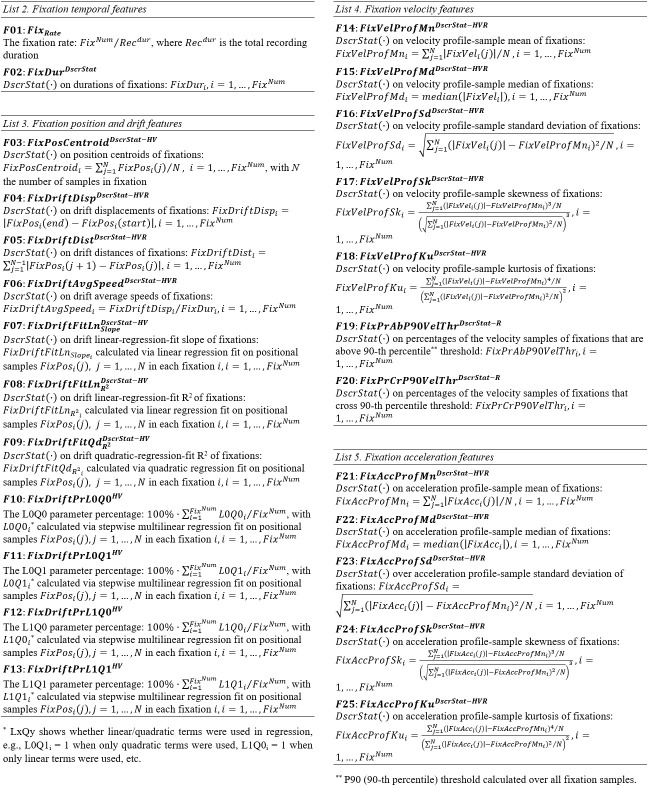


### Saccade Features

The saccades are very fast movements rotating the eyes from one position of focus to another. The peak velocities of saccades can reach over 600°/s. During the initiation of a saccade, the saccade-generating neural circuitry makes an estimation of the difference between the starting and target positions and sends sequences of neural guiding pulses to the extra-ocular muscles to rotate the eye. If the intended target is not accurately reached, one or more small corrective saccades are performed to transfer the eye to the final target position. In next subsections, we present a large variety of features that can be extracted from saccades. Prior to the extraction of saccade features, we post-process the data by filtering out any saccades with durations larger than 70 ms and radial sizes larger than 8° (the adjacent post-saccadic oscillations are filtered out as well). This procedure is performed to avoid any large outliers that can skew the distributions of saccade features, given that during reading usually relatively small saccades are performed. An exception on this post-processing rule was made for features *S49* to *S52*, since the exact role of these features is to measure the frequency of occurrence of such large saccadic events.

#### Features of saccade temporal characteristics

Two basic temporal features of saccades are their duration and rate (*S01*-*S02*, *List 6*). During reading for example, saccade durations usually are in range of 20-40 ms. The incorporation (or not) of saccadic durations when analyzing eye movement data is an important consideration in studies of cognitive processing (33) and during tasks of perceptual selection in human-computer interaction (34). Also, atypical values of saccade temporal features (e.g., larger than usual durations) can be signaling the onset of neural disorders (35), whereas increased saccadic rates have been reported in studies of behavioral disorders like autism (36).

#### Features of saccade amplitude and curvature

**Figure 3.**
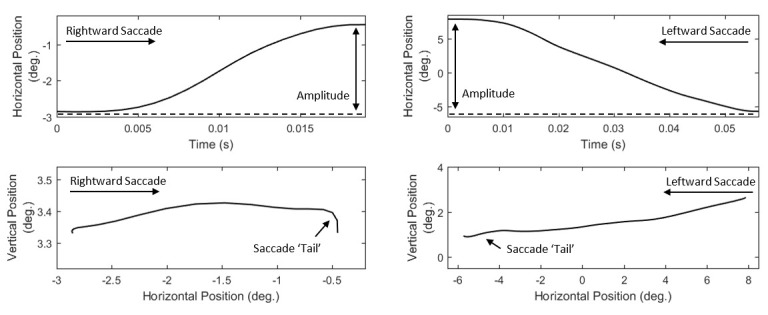
Examples of saccade trajectories in time domain (top panel) and in 2-D plane (bottom panel).

The amplitude of saccades (*S03*, *List 7*) is frequently used as the basic feature to describe their size. The amplitudes of saccades are related to the respective durations and peak velocities (37), giving the opportunity of co-examination of these characteristics. Although the amplitude can provide a basic description of the overall size of a saccade, it cannot describe the curvature characteristics of saccadic trajectories. The modeling of saccade curvature can be important for behavioral studies, given the observed connections of curvature with the distractor-related modulation of eye movements (38). The representation of saccade curvature has been thoroughly reviewed in (39), where a large variety of curvature features (old and new) were described. We have included these features in the current set (*S08* to *S19*, *List 7*) along with additional features that can model the non-linearity of saccade trajectory (*S04*-*S05*, *List 7*). Based on our observation that the ending parts of saccades often show larger degree of non-linearity, we also present two more features for modeling the saccade ending parts (‘tails’) (*S06*-*S07*,* List 7*). In *Figure 3*, we show examples of saccade trajectories both in time domain (position profile) and in 2D-space domain.

#### Features of saccade velocity and acceleration

**Figure 4.**
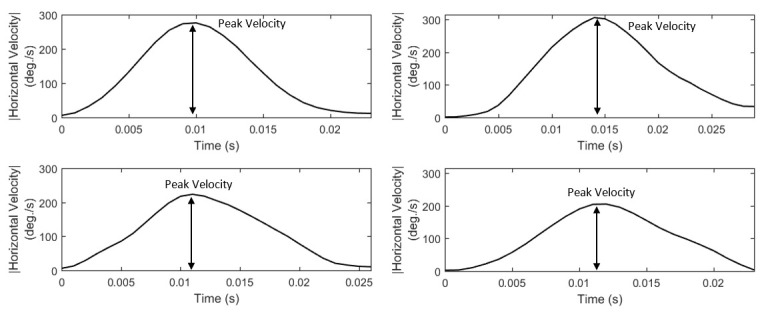
Examples of saccade velocity profiles showing the differences in their peak values and overall shapes.

Saccades are considered to be of ballistic nature and it is assumed that their velocity cannot be modulated intentionally (40). Thus, the dynamic features of saccades provide a valuable source for exploring the background neurophysiological activity. In previous studies, the characteristics of saccadic velocity have been investigated as indicator of (de-)activation (41) and arousal (42). In *Figure 4*, we can observe examples of the characteristics of various saccade velocity profiles. A prominent feature that can be extracted easily from velocity profiles is peak velocity (*S21*,* List 8*). Additionally, as previously done for fixations, we extract a set of profile-modeling features by employing descriptive statistics to represent the overall ‘shape’ properties of velocity profiles (*S22* to *S26*,* List 8*).

**Figure 5.**
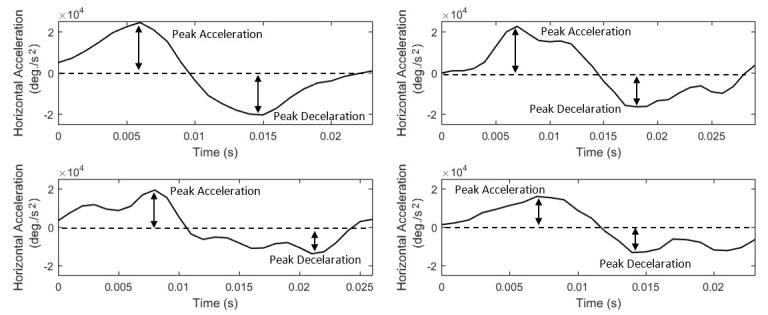
Examples of saccade acceleration profiles showing the differences in their peak values, durations, and shapes of the acceleration-deceleration phases.

The acceleration of saccades is directly related to the underlying forces moving the eyeball. Thus, saccadic acceleration can provide important information related to the dynamic properties of eye movements. The existence of asymmetries in the shapes of saccadic acceleration-deceleration phases has been previously reported in (43), and it has been shown that the characteristics of these phases can be modulated by motor learning (44). Also, abnormal characteristics of the saccade acceleration-deceleration phases have been reported in studies of autism spectrum disorders (45). In *Figure 5*, we show examples of saccade acceleration profiles (for the same saccades as in *Figure 4*) demonstrating the peaks, durations, and shapes of the acceleration-deceleration phases. To describe the basic properties of saccadic acceleration, we extract features for the peak values of acceleration and deceleration phases (*List 9*,* S27*-*S28*), and also, we extract acceleration profile-modeling features via the application of descriptive statistics (*List 9*,* S29* to *S33*).

#### Features of saccade-characteristic ratios

Features that represent ratios of saccadic characteristics can provide valuable clues for the inter-connections of oculomotor mechanisms. Also, such features can be used to provide robustness against exogenous effects when such effects are not desired (e.g., effects of stimulus layout). Various ratio features have been investigated in the past in studies of Parkinson’s disease (peak velocity-mean velocity ratio or Q-ratio) (46), as indicators of alertness (peak velocity-duration ratio or Saccadic-ratio) (47), and in biometrics (peak acceleration-peak deceleration ratio) (48). In *List 10*, we present the extracted saccade-characteristic ratio features (*S34* to *S40*).

#### Lists of Saccade Features

**List 6. - List 8.**
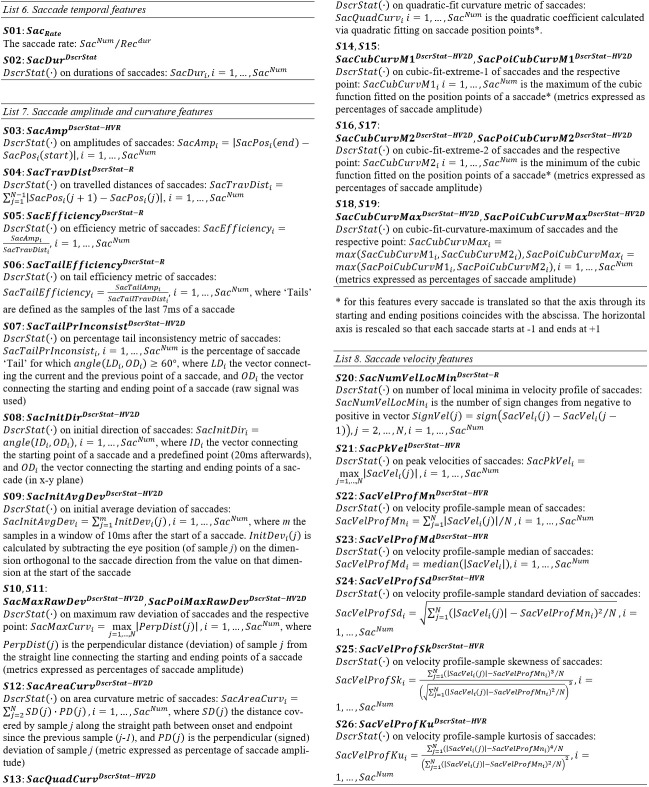


**List 9. - List 12.**
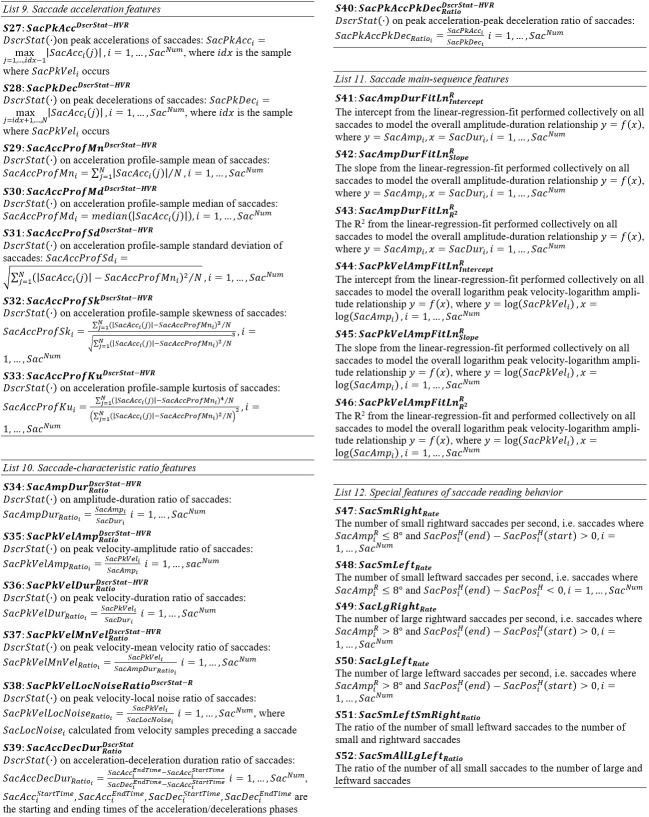


**Figure 6.**
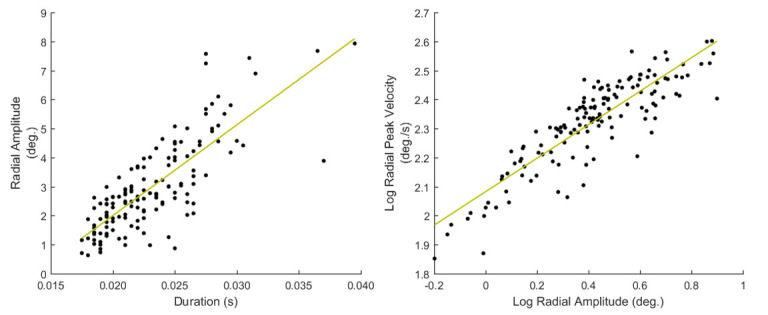
Saccade main-sequence relationships and the respective linear regression fits for amplitude-duration (left) and the logarithms of peak velocity-amplitude (right).Features of saccade main-sequence characteristics

#### Features of saccade main-sequence characteristics

A more sophisticated way to describe the relationships between basic saccadic characteristics is to create a collective model (e.g., via curve fitting) using all the saccadic event instances in a recording. In the work of (37) the general relationships of feature-pairs of amplitude-duration and peak velocity-amplitude were investigated, and the term ‘main-sequence’ (borrowed from Astronomy) was used to describe them. The properties of main-sequence have been investigated in connection to mental workload and arousal (42 49, ), and also, they have been employed for modeling saccadic vigor in eye movement biometrics (48). The features extracted to describe the main-sequence relationships (*S41*to *S46*, *List 11*) were modeled by fitting linear curves directly on the amplitude-duration data and on the logarithms of peak velocity-amplitude data (due to non-linear relationship). In *Figure 6*, we show examples of the performed fitting on the main-sequence data.

#### Special features of saccade reading behavior

Due to the use of reading paradigm, we also extracted a specialized group of saccadic features (*S47* to *S52*, *List 12*) that combine amplitude and direction cues, and they can potentially represent saccadic events connected to the reading behavior of subjects, e.g., forward read words, corrections and word regressions, line changes etc. It should be mentioned that previous research specialized on the study of eye movements during reading has outlined the importance of similar features and identified possible sources of their variability (3). The modeling ofthe extracted features into more complex entitiesspecifically related to reading behavior is out of the scope of our work, however, in section *Limitations and Further Extensions*we discuss about several studies that can be useful during the implementation of such complex features.

### Post-saccadic Oscillation Features

A post-saccadic oscillation is a small oscillatory movement that can occasionally appear after a saccade. The term post-saccadic oscillation can be used to cover movements appearing in various manifestations, e.g.,as small rapid movements, known as dynamic overshoot (50), or as slower and smoother movements, known as glissadic overshoots(51). Although, there are several studies that relate the appearance of glissadic phenomena with fatigue (52) and idiosyncratic characteristics (50), the exact sources and the role of post-saccadic oscillations is not yet fully understood. Also, their recording has been found to be pronounced for specific eye-tracking technologies (53) and influenced by filtering.

#### Features of post-saccadic oscillation temporal characteristics

The basic features that are extracted to model the temporal characteristics of post-saccadic oscillations are the duration (*P01*) and two features modeling the frequency of appearance of post-saccadic oscillations, the interval between post-saccadic oscillations and the percent of saccades followed by a post-saccadic oscillation (*P02*, *P03*, *List 13*). To further quantify the frequency of appearance of different manifestations of post-saccadic oscillations we extract features that quantify the percentages of slow, moderate, and fast post-saccadic oscillations (*P04*, *P05*, *P06*, *List 13*). The thresholds for the categorization of post-saccadic oscillations into slow, moderate and fast were selected after careful examination of their characteristics during the pre-processing stage.

#### Features of post-saccadic oscillation shape

**Figure 7.**
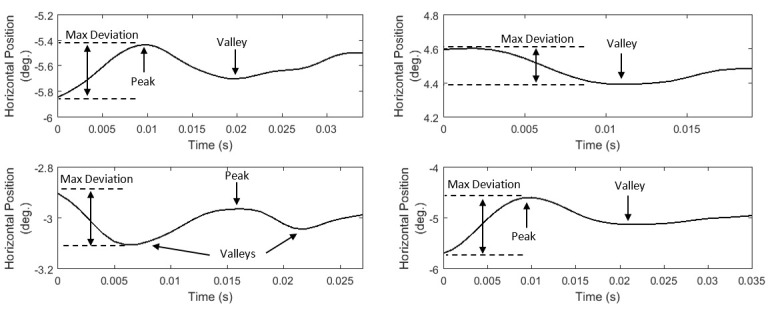
Examples of post-saccadic oscillation position profiles showing the differences in their ‘oscillatory’ shapes.

Instead of extracting the ‘amplitude’ of post-saccadic oscillations (absolute difference between starting and ending positions), which is probably less informative due to their ‘oscillatory’ shape, we extract a feature that represents the maximum absolute deviation in these ‘oscillatory’ shapes (*P07*, *List 14*). We further model the position profiles of post-saccadic oscillations by extracting two features that represent the number of local minima (valleys) and maxima (peaks) (*P08*, *P09*, *List 14*). In *Figure 7*, we present examples of post-saccadic oscillation position profiles demonstrating the variability in their ‘oscillatory’ shapes, and showing the features that can be extracted to model this variability.

#### Features of post-saccadic oscillation velocity and acceleration

**Figure 8.**
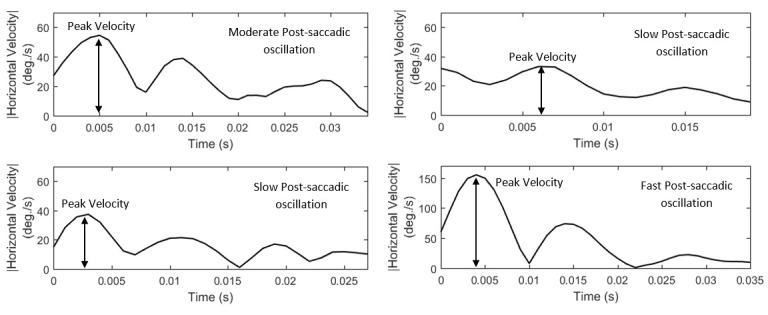
Examples of post-saccadic oscillation velocity profiles showing the differences in their peak velocities and shapes.

**Figure 9.**
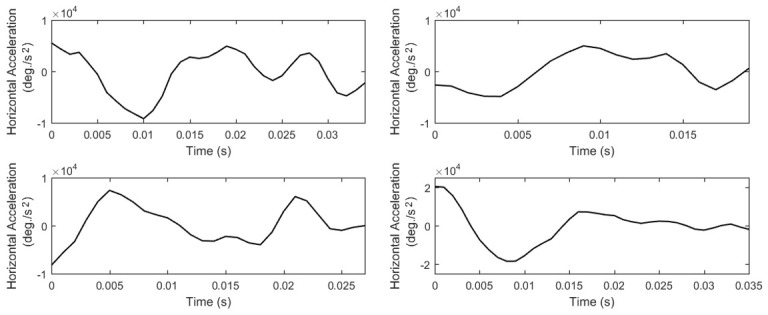
Examples of acceleration profiles of the previously shown post-saccadic oscillations.

Due to the nature of post-saccadic oscillations their velocity profiles usually have multiple peaks. We extract the feature of peak velocity (*P10*, *List 15*) from the largest of them –most times it is the first peak. In *Figure 8*, we show examples of velocity profiles of post-saccadic oscillations (for the same events as in *Figure 7*) and demonstrate the differences in the peak velocities of fast, moderate, and slow post-saccadic oscillations. In *Figure 9*, we present the respective acceleration profiles. As was performed for fixations and saccades, we extract a set of features that model the ‘shapes’ of velocity and acceleration profiles of post-saccadic oscillations via the useof descriptive statistics (*P11* to *P20*, *Lists 15-16*).

#### Features of saccade/post-saccadic oscillation characteristic ratios

Given the fact that every post-saccadic oscillation can be tied to a preceding ‘parent’ saccade, we extract three additional categories of features that can be used to model the possible interrelationships between the characteristics of saccades and their adjacent post-saccadic oscillations. These features (*P21* to *P24*, *List 17*) are extracted by computing the ratios between important characteristics of post-saccadic oscillations and saccades, specifically, duration, amplitude (saccade) or deviation (post-saccadic oscillation), and peak velocity.

## Experiments

### Subjects

The experiments were performed with the participation of 298 subjects (162 males/136 females) with ages from 18 to 46 years, (M = 22, SD = 4.3). All subjects had normal or corrected vision (151 normal / 147 corrected with 61 glasses / 86 contact lenses) and filled a questionnaire to verify that they did not have any recent severe head injury that could affect the oculomotor functionality. The study was approved by the institutional review board of Texas State University and the participants provided signed informed consent.

### Apparatus and Recording Setup

The eye tracking system used for the experiments was an EyeLink 1000 eye tracker with a sampling rate of 1000 Hz. The eye tracker operated in monocular mode capturing the left eye. The typical vendor specifications of this system report accuracy of 0.5° and spatial resolution of 0.01° RMS. In our experiments, we followed a strict protocol to ensure the high quality of recordings by restricting the allowed calibration accuracy error to maximum values lower than 1.5° and average values lower than 1°. We practically measured the average calibration accuracy over all recordings to be 0.48° (SD = 0.17°) and the average data validity to be 94.2% (SD = 5.7%). Validity is defined as the percentage of samples that were successfully captured by the eye-tracking device during a recording. Common sources of failure to capture (invalidity) can be blinks, moisture, squinting etc. During the recordings, each subject was comfortably positioned at a distance of 550 mm from a computer screen with dimensions 474 × 297 mm and resolution 1680 × 1050 pixels, where the visual stimulus was presented. To mitigate any possible eye-tracking artifacts from small head movements, the subjects’ heads were stabilized using a chin-rest with a forehead.

#### Lists of Post-saccadic oscillation Features

**List 13. - List 17.**
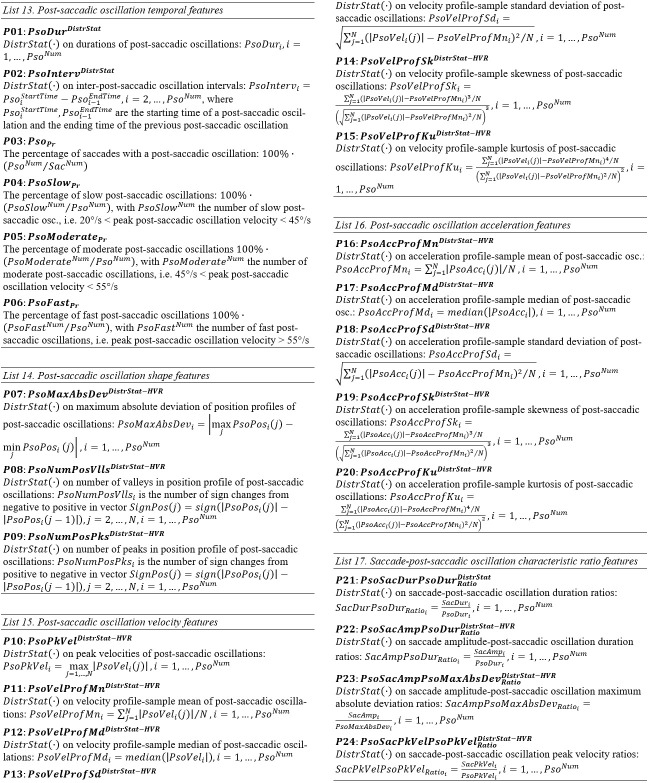


## Experimental Paradigm

The data used in this study are from a text reading experiment, where every subject performed two recordings in two sessions separated by an interval of 13 to 42 minutes (M = 19.5, SD = 4.2). Between these two sessions the subjects performed other eye movement tasks (non-text stimuli) and had brief periods of rest to mitigate eye fatigue. The visual stimulus of the text reading experiment consisted of excerpts from the poem of Lewis Carroll “The Hunting of the Snark”. The first six stanzas were presented in first session and the next six stanzas in second session. The text excerpts were presented in white color on a black background using Times New Roman bold font of size 20pt, single-spaced, corresponding to line height of 0.92° on the presentation screen. Screenshots of the used visual stimulus are provided at:https://digital.library.txstate.edu/handle/10877/6904. The participants were asked to silently read the text, and the totally given time was 60 seconds. The recordings were post-processed to extract the parts that corresponded to the first full pass of the text by each subject.

## Analysis Methods and Results

### Methods for the Assessment of Central Tendency, Variability, and Reliability

In this section, we present the performed analysis for the exploration of the central tendency, variability, and test-retest reliability of the extracted features. For the calculation of summary statistics describing the central tendency and variability of features we employ the median and inter-quartile range over the feature values from the experimental population. We use these measures instead of the mean and standard deviation because these measures are expected to summarize the feature values more robustly both for normally and non-normally distributed features. We also employ two powerful measures for assessing reliability, the Intra-class Correlation Coefficient(ICC) and Kendall’s coefficient of concordance (Kendall’s W), used for normally (the former) and non-normally (the latter) distributed features (see next section for definitions). In our experimental paradigm, the assessment of reliability is particularly important because it can serve two purposes: a) it reveals the stability of measurements taken on different occasions (sessions), and b) it can be used as a proxy of the relative variability of feature values between subjects (inter-subject) and sessions (intra-subject), thus indicating the relative discriminatory power of each feature.

#### Assessment of normality

To assess the direct normality of features we employ the Pearson’s*χ^2^* test and calculate the *p* values at 5% significance level. Features that are not directly normal are subjected to a number of classic normalization transformations followed by reassessment of the test. The used transformations applied on each feature distribution *X* are: the logarithm →log⁡(X+1), the square root → sqrt(X+0.5), the cube root → sign(X)∙^3^√X, the reciprocal →1/X, the arcsine → 2∙sin^-1^⁡X (for proportions), and the logit → log⁡adjX/(1-adjX) (for proportions and features in range [0, 1]) with adjX representing *X* adjusted in range [0.025, 0.075] to avoid undefined cases. The logarithm, square root, cube root, and reciprocal were complementary evaluated for the reflection transformation → max(X)+1-X. Furthermore, in order to evaluate cases where the deviation from normality is due to outliers at the extremes, we perform Winsorization (54) with maximum-minimum limits at 5%-95% percentiles of distribution, and we reassess normality following the previous procedures.

#### Assessment of reliability

**Intraclass Correlation Coefficient (ICC)**: The ICC is a measure that can be calculated for normally distributed data in order to evaluate either the absolute agreement (accounts for systematic differences) or the consistency (does not account for systematic differences) of quantitative measurements, and thus, it can be used to assess the reliability among different occasions (in our case different recording sessions). Six basic forms of ICC and their calculation procedures are described in (55). In the current work we use the ICC to assess absolute agreement, and for this reason, we employ the third ICC form (denoted as ICC(2, 1) in (55)). The original approach for calculating the respective variance estimates is based on ANOVA tables. For cases involving two-way random effect models (like the current case) there is also a more robust approach for this calculation based on variance component maximum likelihood (VCML) analysis (56). We currently adopt this approach to calculate the ICC. The ICC takes values in range [0.00, 1.00] (1.00 indicates complete agreement). The work of (57) suggested some rules of thumb for interpreting the ICC values, in specific, [0.75, 1.00] indicates ‘excellent’ agreement, [0.60, 0.75) indicates ‘good’ agreement, [0.40, 0.60) indicates ‘fair’ agreement, and [0.00, 0.40) indicates ‘poor’ agreement.

**Kendall’s coefficient of concordance (Kendall’s W):** The Kendall’s W (58) is a non-parametric measure that can be used to assess occasion agreement without the requirement for normally distributed data. The Kendall’s W is calculated as a normalization of the Friedman test statistic (59) in range [0.00, 1.00], with 1.00 indicating complete agreement. The process of estimating Kendall’s W does not make any prior assumption for the nature of the data distribution but instead performs statistical calculations based on data rankings. Since Kendall’s W is calculated for non-normally distributed data and since there are no similar rules (ranges) for the interpretation of values as for the ICC (‘excellent’, ‘good’ etc.), it is not advised to directly compare values of ICC and Kendall’s W.

### Results and Discussion for Central Tendency, Variability, and Reliability

The tables of results presented below are structured in two-levels: the top part presents results for *single-value features*. The bottom part presents results for*multi-value features* (six feature subtypes are presented in corresponding columns). As already described, these multiple values are extracted by calculating descriptive statistics (columns *Mn*,* Md*,* Sd*,* Iq*,* Sk*, *Ku*) onvalues from multiple feature instances in a recording. The tables present values only for the independent components of eye movement (horizontal *H*, and vertical*V*), except for the features extracted only from the radial component or from trajectory in 2-D plane. In *Tables 1*,* 3*,and* 5* (fixations, saccades, and post-saccadic oscillations respectively) we present the values of central tendency (median, denoted MD) and overall variability (inter-quartile range, denoted IQ) for features values across subject population. In *Tables 2*,* 4*,and* 6* we present the respective measures from the assessment of normality and reliability of features. In this case, for each featurethere is one column indicating the maximum p value (p) calculated following the described procedures for normality assessment, and the adjacent column presents the value of either the ICC when p value denotes a normally distributed feature (p ≥ 0.05), or Kendall’s W when p value denotes a non-normally distributed feature (p < 0.05). To further facilitate the overview of results, the cells that correspond to non-normal features have been highlighted using light-grey shading. Although the two reliability measures (ICC/W)are presented interchangeably in the same column for simplicity, we should once more emphasize that it is not advised to directly compare their values.

In *Table 1*, we can overview the typical values of fixation features calculated over the experimental population. We can observe that the median fixation duration was calculated to be about 200 ms (*F02*) and corresponds on an average rate of about 3-4 fixations per second (*F01*). This duration is within the expected range for fixations during reading, and similar values have been reported in previous research studies (3 19, ). Since the fixation centroid (*F03*) is a direct measure of position, the extracted values for this feature are heavily affected by the positioning and centering of the stimulus. However, when a common stimulus is used for all subjects (as in our experiments) the median and inter-quartile range can provide clues about the existence of systematic error and its variability, either system-related or subject-related (unique error signature) (28). As revealed from the values of *F05*-*F06*, the drift during fixation affects in a similar way both components of eye movement. Furthermore, the drift speeds of the two components (*F06*) seem to be very close to previously reported values of 0.5°/s (24). The values of the drift linear-fit slope feature (*F07*) reveal a positive tendency for the horizontal component and negative tendency for the vertical. Another important observation is that the values of the quadratic-fit R2 feature (*F09*) are larger than these of the linear-fit R2 feature (*F08*), which seems to indicate the occasional appearance of non-linearity (curvature) in fixation drifts (see *Figure 1*), a phenomenon previously reported in (60). Finally, the calculated values for velocity and acceleration (*F14 to F25*) demonstrate the relatively low levels of eye mobility during fixations, compared to the corresponding levels for saccades and post-saccadic oscillations (see corresponding tables).

**Table 1.**
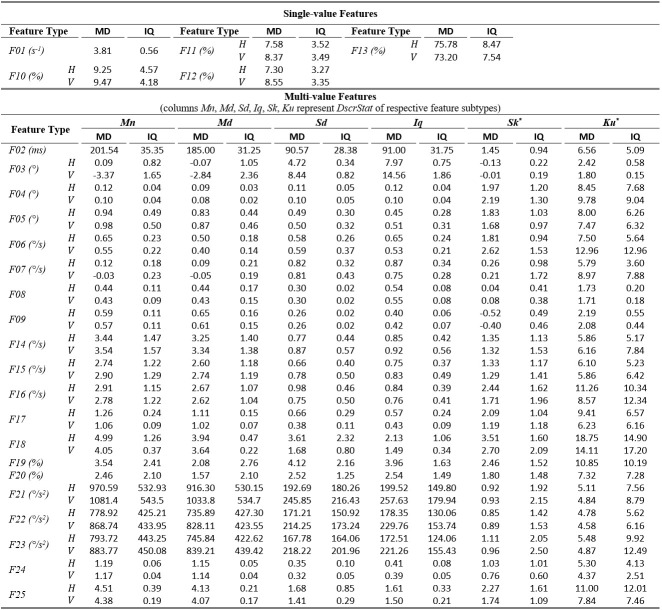
Statistics of central tendency and variability for fixation features over the experimental population.

*Skewness and kurtosis are unit-less measures, so, the feature units do not apply on them

The examination of *Table 2* allows for an assessment of the normality and reliability of fixation features. In overall, 50.7% of fixation features (feature subtypes) are found to be normally distributed and the rest are distributed non-normally. An examination of the shaded parts of the table (non-normal features) reveals that there is a general tendency for non-normality from the acceleration feature categories, and whenusing the kurtosis (*Ku*) descriptive statistic irrespectively of feature category. For the case of fixations, the calculated ICC values for assessing reliability are in range of 0.06 to 0.92. Following the categorization suggested in(57) we can see that 32.5% of them are in the region of ‘excellent’ reliability, 20.5% in the region of ‘good’ reliability, 23.9% in the region of ‘fair’ reliability, and 23.1% in the region of ‘poor’ reliability. The top performing fixation features in terms of reliability are* F14*,*F15*,*F16* (modeling of fixation velocity profile with mean, median, and standard deviation), *F09 *(R2 when modeling fixation drift with quadratic-fit), and *F02* (fixation duration). For the case of non-normal features, the calculated W values are in range of 0.52 to 0.98. The difference in ranges of ICC and W (values of W seem to becompressed in the upper half of range [0.00, 1.00]) portrays the risk of attempting to directly compare the values of the two measures.

**Table 2.**
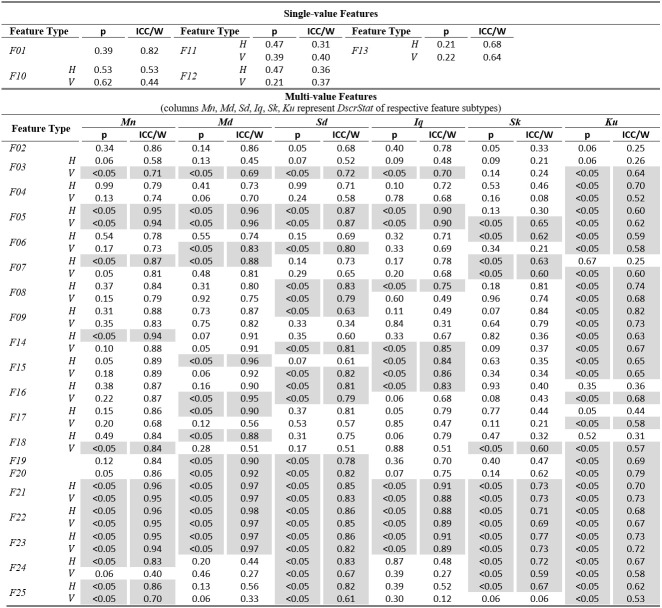
Statistics of normality and reliability for fixation features over the experimental population.

From the respective values we can see that the top performing fixation feature categories based on Kendall’s W measure are *F21*, *F22*,*F23* (modeling of fixation acceleration profile with mean, median, and standard deviation), and *F05* (travelled distance during fixation drift). It is interesting to observe that although the eye mobility is relatively limited during fixations, the dynamic features (based on velocity and acceleration) seem to provide the best test-retest measurement agreement both for the case of normal and for non-normal features.

In *Table 3*, we present the values for the features extracted from saccades. The median duration (*S02*) over the experimental population was calculated to be about 28 ms. This duration seems to be justified given the relatively small amplitude of the saccades performed during reading, and it is within the range reported in other studies that employed the reading paradigm (19 61, ). The median rate of saccades (*S01*) is similar but slightly lower than the rate of fixations, possibly due to the post-filtering of large saccadic events. A very interesting group of features are those that model the curvature of saccadic trajectory. As explained, the feature of saccade efficiency (*S05*) models the difference between the amplitude and the actual travelled distance during a saccade. The smaller values of saccade tail efficiency (*S06*) (efficiency at the ending part of saccade) when compared to overall saccade efficiency (*S05*) indicates the appearance of ‘hooks’ in saccade trajectory towards the ending part (when the post-saccadic oscillation phase begins). Qualitative observations of such phenomena have been reported in previous studies (62). The calculated value for the point of maximum raw deviation (*S11*) shows that in general the maximum raw deviation can be expected to occur around the middle (54%) of saccadic trajectory. Since the horizontal component of eye movement is typically more active during the reading task, the values for the dynamic features are much larger than for the vertical component. The median horizontal peak velocity (*S21*) was calculated to be about 170°/s, and the relatively large values of the *Sd* and*Iq* feature subtypes reveal a considerable variability of the peak velocity during the duration of a recording. The values of peak acceleration and deceleration (*S27*, *S28*) are both close to 13000°/s2. Similar values but for much smaller population are reported in (61). The median peak acceleration appears to be in overall slightly larger than the peak deceleration, however, the reported variability does not allow to support the generality of this phenomenon. The calculated values for the features of acceleration-deceleration duration ratio (*S39*) and peak acceleration-peak deceleration ratio (*S40*) also suggest the volatility of this difference. The median acceleration-deceleration duration ratio seems to be slightly over one although it is expected that the larger values of peak acceleration (compared to peak deceleration) should correspond to smaller values of duration. An explanation for this discrepancy is that, in general, there is greater difficulty to accurately estimate the exact durations of the acceleration-deceleration phases (atypical profiles, multiple zero-crossings etc.) compared to the estimation of peak values. The overview of the features of saccadic reading behavior further clarifies the previously discussed difference in fixation and saccade rates (features *F01*, *S01*). In specific, by adding the rates of ‘large’ saccades (*S49*, *S50*) and ‘small’ saccades (*S47*, *S48*) we get a value that is much closer to the fixation rate. The rate of leftward large saccades (*S50*) is 0.4 (about one such saccade per two seconds), and seems to be consistent with the expected rate of line changes during normal reading. The calculated value for the rate of leftward small saccades (*S48*) is 0.8 (about one such saccade per second), a value that seems to be quite large to represent only word regressions. This value can be attributed to small corrective saccades performed during reading, e.g., for correcting undershoots during line changes (3).

**Table 3.**
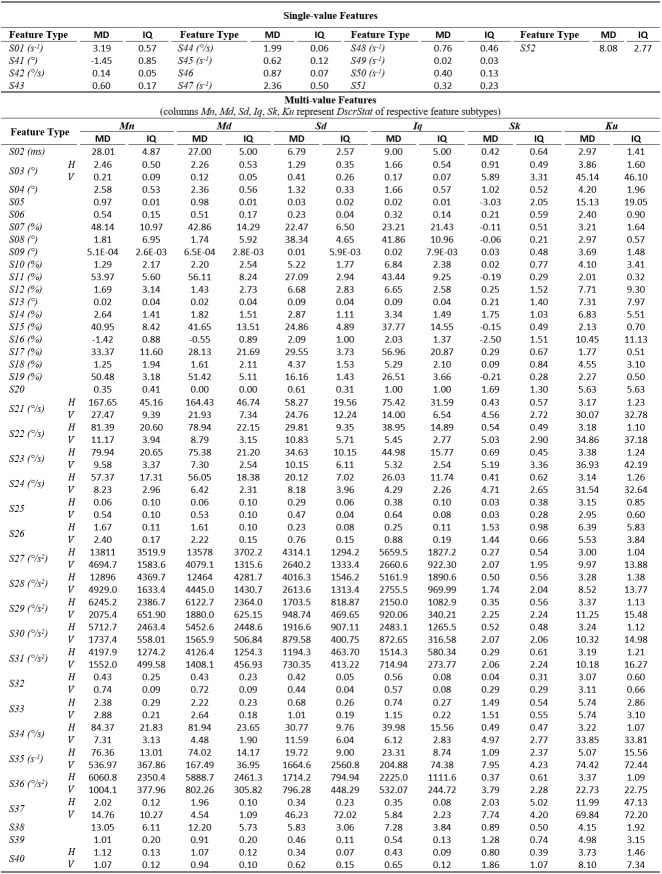
Statistics of central tendency and variability for saccade features over the experimental population.

The overview of the results from assessing the normality and reliability of saccade features is provided in *Table 4*. An initial observation is that the percentage of saccade features that are normal (or can be normalized) is much larger (74%) than previously. A prominent clustering of non-normal features seems to occur for some of theskewness (*Sk*) and kurtosis (*Ku*) feature subtypes. Also, a considerable clustering of non-normal features can be observed in feature categories *S05*,* S06*,*S07* (saccade efficiency, tail efficiency, tail inconsistency). For the case of saccade features, the calculated ICC values range from 0.00 to 0.96, with relatively larger percentage (42.1%) of them being highly reliable (‘excellent’ reliability), 19.9% are considered of ‘good’ reliability, 16.9% present ‘fair’ reliability, and 21.1% present ‘poor’ reliability. The saccade feature categories with the top values of ICC are *S36 *(the ratio of saccade peak velocity to saccade duration), *S29*,* S30*,*S31* (modeling of saccade acceleration profile with mean, median, and standard deviation), and *S06* (saccade tail efficiency). Top values refer to horizontal (or radial) components since they are more reliable than vertical components. There are also several other feature categories with exceptional reliability (ICC > 0.9), as for example *S02* (saccade duration) and *S27*-*S28* (peak acceleration and peak deceleration). As previously, the calculated Kendall’s W values for the non-normal features seem to be compressed at the upper half of range, varying from 0.44 to 0.98. The excellent reliability of feature *S36* (ratio of saccade peak velocity to saccade duration) is further solidified by the higher Kendall’s W measure calculated for the *Mn* subtype of this feature, which was designated as non-normal (the rest subtypes were designated as normal). The same holds for features* S06* (saccade tail efficiency) and *S02* (saccade duration) for subtype*Md*. These and other similar cases (where some feature subtypes are designated as normal and some as non-normal) seem to imply that although the values of ICC and Kendall’s W cannot be directly compared, there is a certain degree of correspondence in their relative assessments about which feature categories are more reliable than others. Finally, another saccade feature category with non-normal memberswith very high W values is *S20* (number of local minima in velocity profile).

**Table 4.**
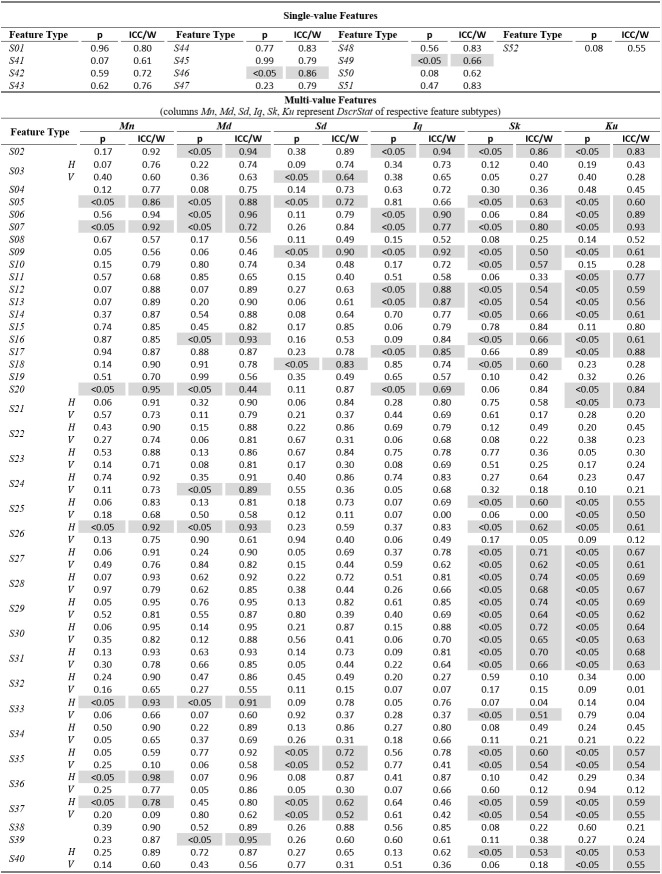
Statistics of normality and reliability for saccade features over the experimental population.

In *Table 5*, we show the values for the post-saccadic oscillation features. The median duration (*P01*) was calculated to be about 14 ms, and the median interval between post-saccadic oscillations (*P02*) was found to be about 400 ms (though, with high variability). In overall, post-saccadic oscillations seem to occur at more than half of the saccades (*P03*). This finding agrees with previous observations (19) and further justifies the necessity for modeling the characteristics of post-saccadic oscillations. The vast majority of post-saccadic oscillations (76.5%) are ‘slow’ (*P04*) (peak velocities between 20°/s and 45°/s), whereas the percentages of ‘moderate’ (*P05*) (peak velocities between 45°/s and 55°/s) and ‘fast’ (*P06*) (peak velocities larger than 55°/s) post-saccadic oscillations are about 11-12% each. The velocity and acceleration profile-modeling features (*P10* to *P20*) demonstrate the intermediate levels of eye mobility compared to saccades and fixations. Also, the examination of the ratio features shows that the post-saccadic oscillations have about 2-3 times smaller duration (*P21*) compared to the preceding saccades, whereas their peak velocities are about 5-6 times smaller (*P24*) than saccades (for horizontal component).

**Table 5.**
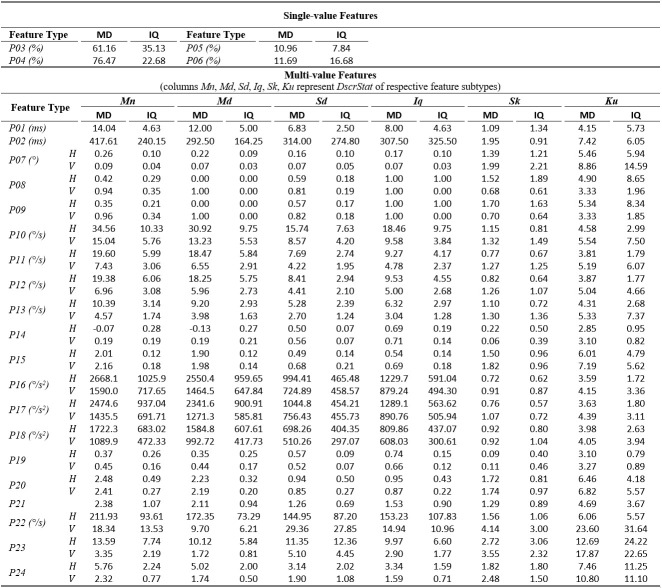
Statistics of central tendency and variability for post-saccadic oscillation features over the experimental population.

An overview of *Table 6* can reveal the characteristics of normality and reliability of post-saccadic oscillation features. The percentage of normal (or normalized) post-saccadic oscillation features is slightly higher than the percentagefor fixations, lying at 54.9%. 

**Table 6.**
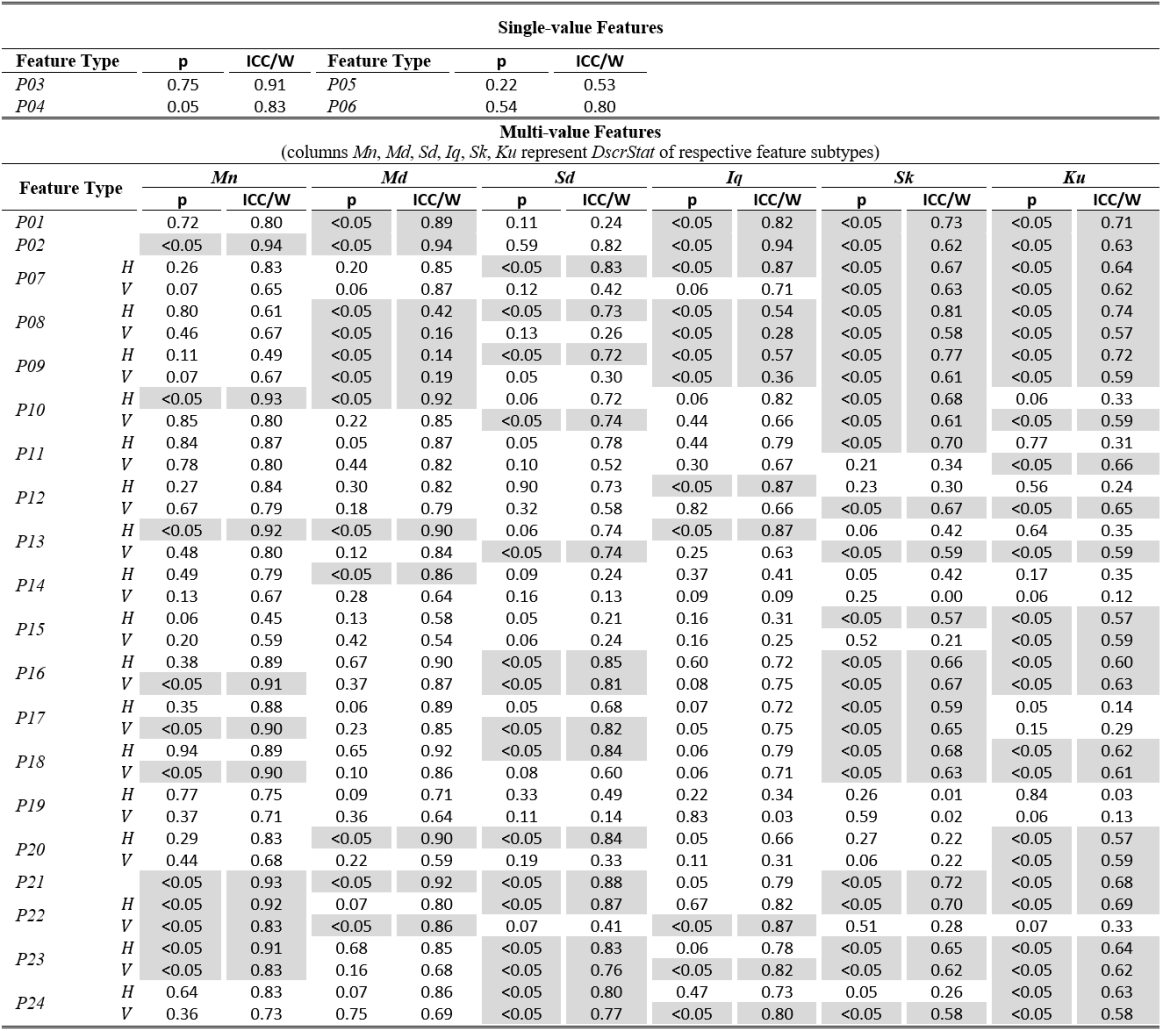
Statistics of normality and reliability for post-saccadic oscillation features over the experimental population.

The ICC values for the case of post-saccadic oscillations range from 0.00 to 0.92, and the corresponding levels of reliability for the post-saccadic oscillation features are 35.5% ‘excellent’, 23.4% ‘good’, 12.1% ‘fair’, and ‘poor’ 29.0%. Among the most reliable categories of post-saccadic oscillation features are *P03* (percentage of saccades followed by a glissade), and *P16* and *P18* (modeling of post-saccadic oscillation acceleration profile with mean and standard deviation). The values of Kendall’s W vary from0.14 to0.94 with the most reliable featuresappearing in categories*P02* (interval between post-saccadic oscillations), *P10* (peak velocity of post-saccadic oscillations), *P13*(modeling of post-saccadic oscillation velocity profile withstandard deviation), *P21* (ratio of durations of saccades and adjacent post-saccadic oscillations), and *P22* (ratio of amplitudes of saccades and durations of adjacent post-saccadic oscillations).

### Factor Analysis Methods and Results

#### Preparation of feature subset for factor analysis

We extracted 101 general categories of features from fixations, saccades, and post-saccadic oscillations.From these categories, the *single-value features* contribute x 1 feature values, and the *multi-value features* contribute x 5 feature values. Also, the features that are extracted from horizontal, vertical, and radial components contribute x 3 feature values, the features extracted from horizontal and verticalonly components contribute x 2 feature values, and the features extracted from radial-only component or from 2D-trajectory contribute x 1 feature values. Thus, by combining the contributions from all feature categories we finally result with 1112 unique feature valuesthat are extracted from each recording of every subject. Not all of these features are suitable for factor analysis, and for this reason, we followed a procedure for the preparation of a subset of features. Our first step was to remove any features that had undefined values or missing data for any subject. On this basis, 3 features were removed. Our second step was to drop any features that were not normally distributed and could not be transformed into normal (following the procedures described in previous section). This left 687 features. The remaining features contained redundant features. For example, some features were based on either the mean (*Mn*) or the median (*Md*) of the same distribution, or the standard deviation (*Sd*) or the interquartile range (*Iq*) of the same distribution. We did not need two estimates of the central tendency of eye-movement feature distributions (mean, and median) so the less reliable (lowest ICC) measure was dropped from further analysis. Similarly, we did not need two measures of variance (interquartile range, and standard deviation) so the less reliable was dropped. After this step, there were 582 features left. We also intercorrelated every feature with every other feature, and found those pairs of features that were intercorrelated (Pearson’s r) greater than 0.90 (absolute value). We considered such pairs of features effectively redundant. The lower reliability feature from each pair was dropped from further analysis. This removal of redundant features was done for all subjects for session 1 data. This left 323 features. At this stage, the correlation matrix of the features was not positive definite, and could not, in this condition, be submitted to a factor analysis. As few highly intercorrelated features as possible were removed to ensure that the feature correlation matrix was positive definite. This left 274 features. In the final step, we removed all remaining features with an ICC less than, or equal to 0.7. This left our final dataset, ready for submission to factor analysis, with 95 features.

#### Factor analysis methodology

**Figure 10.**
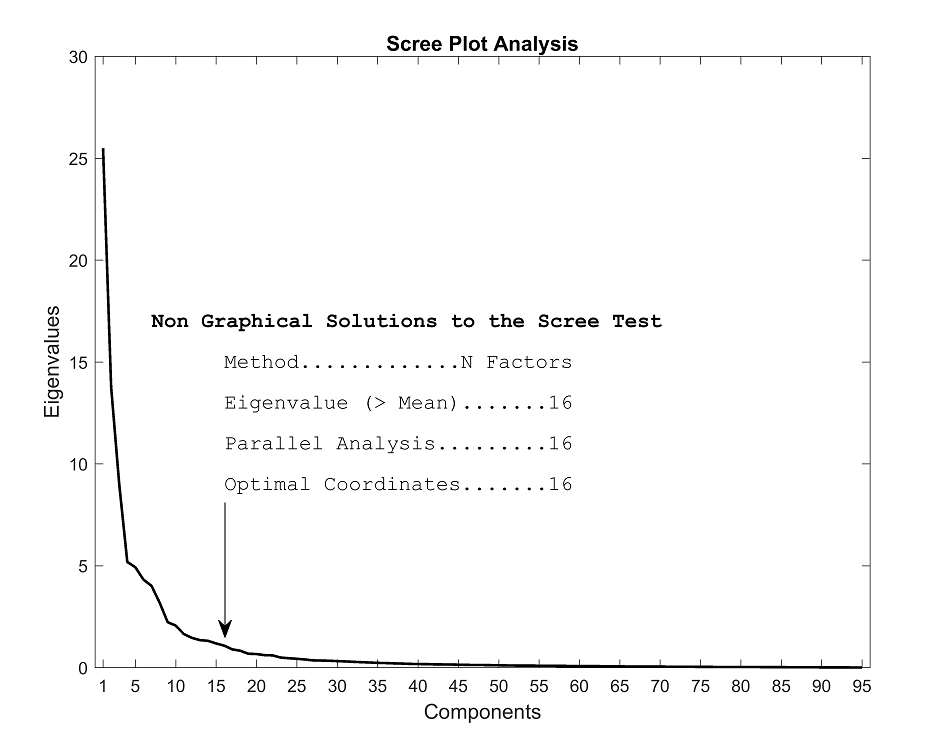
Scree plot to determine the number of factors to look for in the final data set. The three more commonly used analysis (mean eigenvalue, parallel analysis and optimal coordinates) all indicate that there are 16 factors.

We used SPSS (IBM SPSS Statistics for Windows, Version 24.0 Armonk, NY: IBM Corp.) to conduct our factor analysis. To determine the number of factors we conducted a scree plot analysis in R, using the package “nFactors” 63), as shown in *Figure 10*. The factor analysis was based on the correlation matrix of the 95 features in the final data set. We searched for 16 factors, based on the scree plot analysis. In our analysis, we employed maximum likelihood extraction, which is well suited to multivariate normal distributions (64). Also, we employed the most widely used varimax rotation to enhance the interpretability of the resulting factors.

#### Factor analysis results

The factor analysis results are presented in *Table 7*. We employed the rotation factor matrix to extract the most important features.For each factor we created a description-name, based on our best interpretation of the meaning of the 4 most heavily weighted features (absolute value).*Table 7* also contains the percent of variance accounted for by each factor. It also lists the 4 most heavily weighted features contributing to that factor as well as the feature weight (absolute value). The “Saccade Speed” factor accounted for 21.3% of the variance in the analysis, approximately more than twice as much as any other factor.Two other factors, “Fixation Drift Speed” and “2-D Saccade Distance Travelled” accounted each for more than 10 % of the variance. Factors 11 to 16 each accounted for less than 2 % of the variance. It should be noted that in some cases the most heavily weighted feature was difficult to be interpreted, and thus, we performed additional analysis and investigated intercorrelations with other features to derive more easily interpretable description-names for the respective factors. For example, for factor 14, the most heavily weighted feature is *S25*(modeling of saccade velocity profile with skewness), horizontal (*H*) component, mean (*Mn*) descriptive statistic. This was found to be very highly correlated with the much more interpretable feature *S37*(ratio of saccade peak velocity to saccade mean velocity), horizontal (*H*) component, median (*Md*) descriptive statistic. So, for this factor we finally selected the description-name “Ratio of Horizontal Saccade Peak Velocity to Mean Velocity”. For factor 15, the most heavily weighted feature is *S25*(modeling of saccade velocity profile with skewness), radial(*R*) component, standard deviation (*Sd*) descriptive statistic. In this case, we investigated cases with high and low values for this feature and observed that the cases with high values corresponded to velocity profiles with several minima and maxima. We verifiedthat there is a strong relationship between this feature and feature*S20*(number of local minima in velocity profile), mean (*Mn*) descriptive statistic, which was initially excluded from factor analysis because it could not be normalized. Thus, we finally selected the description-name “Tendency for Multipeaked Saccade Velocity Profiles” for this factor.

**Table 7.**
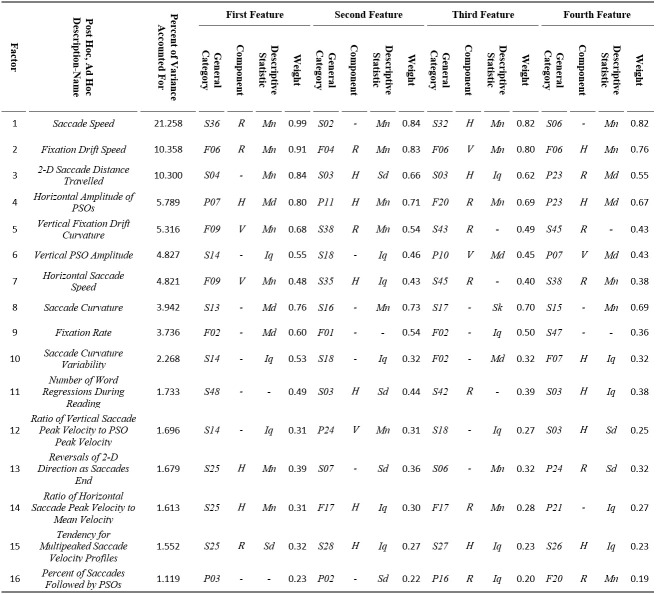
Results from factor analysis showing the most heavily weighted features and the percent of accounted variance for each factor, along with the interpretation of factors via respective Post Hoc, Ad Hoc assigned description-names.

## Limitations and Further Extensions

The current research should be considered within the scope of certain limitations. First of all, the recordings of eye movements were conducted using a specific model of a high-grade eye-tracker (EyeLink 1000). It would be very interesting to investigate the stability of feature values and the results from reliability assessment for eye movement recordings captured with eye-tracker models of different specifications. Since most of the highly reliable features were found to be dynamic and related to velocity/acceleration traces, which are usually the most prone to error, it would be useful to assess the effects of different error sources (eye-tracker dependent etc.) on the features. Second, the current test-retest interval can be considered relatively small (30 min.). Although such an interval justifies a preliminary test-retest analysis for assessing the reliability of features coming from different subjects, the examination of eye movement feature values for larger time intervals is expected to shed light on their long-term stability.

A further extension of the current study involves the combination of the extracted features into more complex entities thatcan be interpreted within the context of cognitive and visual behavior models. Examplesof such models specificallyfor the task of reading have been presented in various previous studies, e.g., see(65), (66), (67), (68), and (69). In orderto implement such more complex entities (complex features) one needsto take into consideration the influencesnot only of fixated word properties but also of previous and upcoming words (lag- and successor-word effects), or it is even possible tocode the features based on larger clusters of words, e.g., word quintets (70).

## Conclusion

In this work, we presented an overview of anextensive collection of features that can be extracted from eye movements. The described features can be used for modeling the characteristics of fixations, saccades, and post-saccadic oscillations,and allow for examining the physical and behavioral properties of eye movements. Alongwith the presented methods for the extraction of features, we examinedtheir variability and test-retest reliability, and performed factor analysis using data from a large population of subjects. The presented methods andanalysis can provide further insights on the temporal, positional, and dynamic properties of eye movements, and can serve as a useful tool forstudies and applications involving the exploration and selection of eye movement features.

### Ethics and Conflict of Interest

The authors declare that the contents of the article are in agreement with the ethics described in http://biblio.unibe.ch/portale/elibrary/BOP/jemr/ethics.html and that there is no conflict of interest regarding the publication of this paper.

### Acknowledgements

This work was supported in part by NSF CAREER grant #CNS-1250718 and NIST grant #60NANB15D325. Special gratitude is expressed to Dr. E. Abdulin and I. S. Vasquez Mondragon for their contribution during the pre-processing of the eye movement recordings.
